# Anatomical changes induced by isolates of *Trichoderma* spp. in soybean plants

**DOI:** 10.1371/journal.pone.0242480

**Published:** 2020-11-16

**Authors:** Camilla Martins Oliveira, Nayane Oliveira Almeida, Mara Rubia da Rocha, Maria Helena Rezende, Renê Gonçalves da Silva Carneiro, Cirano José Ulhoa

**Affiliations:** 1 Departamento de Bioquímica e Biologia Molecular, Universidade Federal de Goiás, Goiânia, Goiás, Brazil; 2 Escola de Agronomia, Universidade Federal de Goiás, Goiânia, Goiás, Brazil; 3 Departamento de Botânica, Universidade Federal de Goiás, Goiânia, Goiás, Brazil; CSIR-National Botanical Research Institute, INDIA

## Abstract

In the current work we evaluated the anatomical changes induced by *T*. *harzianum* and *T*. *asperellum* in two soybean cultivars, BRSGO Caiaponia and NA 5909 RG. Soybean production represents a growing market worldwide, and new methods aimed at increasing its productivity and yield are constantly being sought. Fungi of the genus *Trichoderma* have been widely used in agriculture as a promising alternative for the promotion of plant growth and for biological control of various pathogens. It is known that *Trichoderma* spp. colonize plant roots, but the anatomical changes that this fungus can cause are still less studied. Experiment was conducted in a greenhouse to collect leaves and soybean roots to perform analysis of growth parameters, enzymatic activity of defense-related enzymes and anatomical changes. It was observed that inoculation of *Trichoderma* spp. caused anatomical alterations, among them, increase in stomatal index at the abaxial leaf surface, thickness of the root cortex, thickness of adaxial epidermis, mean diameter of the vascular cylinder, thickness of the mesophyll, and thickness of the spongy parenchyma of the soybean plants. These results indicate that the alterations in these factors may be related to the process of plant resistance to pathogens, and better performance against adverse conditions. This study demonstrates that the anatomical study of plants is an important tool to show the effects that are induced by biological control agents.

## Introduction

Soybean [*Glycine max* (L.) Merr.] is currently the most important oilseed crop in the world due to its economic and nutritional significance, and due to its ever- increasing production rates. In Brazil, the cultivated area corresponds to 36 million hectares, with a productivity of 123 million tons in the 2019/2020 crop [1]. However, the productivity of soybean plants may be limited by diseases caused by phytopathogenic fungi [2]. One of the alternatives proposed for the management of such diseases is the biological control using microorganisms. Among the biological control agents, *Trichoderma* species, such as *T*. *harzianum*, *T*. *asperellum* and *T*. *viride* have been extensively studied regarding the biological control of many diseases worldwide [3, 4]. These species have an arsenal of direct and indirect mechanisms of action against fungal pathogens, including competition by nutrients, mycoparasitism and antibiosis [5–7].

Interactions between *Trichoderma* spp. and plants usually occur at the level of the rhizosphere, where the fungi colonize both epidermal and cortical parenchyma cells of the roots [5]. Once contact has been established, elicitors produced and released by *Trichoderma* spp. induce further plant defenses, such as tissue lignification, production of antimicrobial compounds and synthesis of defense-related proteins [4, 6, 7]. Following induction of the biochemical mechanisms, the changes in plant metabolism are imprinted into the plant structure, and the anatomical characterization of plant organs may highlight structural peculiarities that evidence plant reactiveness to the presence of microorganisms such as *Trichoderma* spp. [8]. It has been reported that the use of *Trichoderma* spp. in association with other plant growth promoting organisms, such as bacteria and fungi, can alter the morphology and physiology of soybean plants [9, 10]. In both studies, the role of *Trichoderma* spp., in morphological and physiological changes in soybeans is not clear.

In this context, the objective of this study was to evaluate structural impacts of the association between *T*. *harzianum* or *T*. *asperellum* and soybean cultivars, by identifying anatomical alterations induced by the fungi, which may improve soybean adaptability and resistance to both biotic and abiotic stresses imposed by the harsh environment of crop fields. The following questions are addressed: (1) Does the treatment with *T*. *asperellum* and *T*. *harzianum* affect the development of NA5909 RG or BRSGO Caiaponia cultivars differently? (2) Do the isolates of *T*. *asperellum* and *T*. *harzianum* affect the defense-related enzymatic apparatus of soybean cultivars? (3) Are there significant anatomical alterations caused by the treatments with *T*. *asperellum* and *T*. *harzianum* that relate to plant adaptability? (4) Which cultivar displays the most desirable anatomical features related to growth performance in the presence of *T*. *asperellum* and *T*. *harzianum*?

## Materials and methods

### Fungal strain and preparation of spore suspensions

*Trichoderma harzianum* (ALL 42) and *Trichoderma asperellum* (T00) were identified at the species level by using the combination of morphological and microbial molecular analysis [11]. The isolates were purified by repeated sub-culturing and stored in 20% glycerol at -80°C in Embrapa Genetic Resources and Biotechnology Collection (Brasilia, Brazil).

Isolates were multiplied in the laboratory, in Erlenmeyer flasks (250 mL) containing grains of parboiled rice previously moistened with autoclaved distilled water (120°C, 40 minutes). The Erlenmeyer flasks were maintained at 25°C under a 12-hour photoperiod for seven days. The spore suspensions were obtained after washing the rice with autoclaved distilled water, and the concentration was adjusted to 5.8x10^9^ viable conidia mL^-1^. A solution of 1% carboxymethylcellulose (CMC), was used to induce adherence of *Trichoderma* spp. spores to the tegument of soybean seeds [12, 13].

### Conditions of plant growth and experimental design

The experiments were conducted under greenhouse conditions, with controlled temperature (21°C to 35°C), and in the laboratory. The experimental design was completely randomized in a 2x3 factorial scheme with six replicates, i.e., two soybean cultivars (NA 5909 RG and BRSGO Caiaponia) and three treatments: 1) Uninoculated control; 2) *T*. *harzianum* and 3) *T*. *asperellum*. Plants were grown in 1.5 L polyethylene pots, containing an autoclaved (120°C, 40 minutes) mixture of sand and soil (1:1, v:v). Seven seeds from each treatment were sown per pot. Additionally, a suspension of viable *Trichoderma* spp. spores (100 mL, 5.8 x 10^9^ spores/mL) was added to the plant pots 18 days after plant germination to ensure the presence of *Trichoderma* spp. in the assay. The plants were measured at seven, fourteen and twenty-one days after sowing (DAS) for comparison of plant height. By the end of the experiment, at 30 DAS, the plants were weighed (fresh weight) and one plant per replicate was collected for the anatomical analyses (n = 6).

### Enzymatic activity in leaves

To estimate the activities of phenylalanine ammonia-lyase (PAL), β-1,3-glucanase (GLU) and chitinase (CHI), five leaves from three plants were collected after 21 DAI and ground with liquid nitrogen using a mortar and pestle. The fine powder (200 mg) was transferred to Eppendorf tubes with 2 ml of extraction buffer containing 10mM Tris-HCl buffer (pH 7), 2 mM EDTA and 150 mM NaCl. The homogenate was centrifuged at 13,000 g for 15 min at 4°C and the supernatant used as the source of enzymes. The enzyme activity was expressed based on protein concentration determined according to the method of Bradford [14] using BSA as a standard. Specific activity was defined as the ratio of enzyme activity (U.ml^-1^) to total protein concentration (mg.ml^-1^) and is expressed in U.mg^-1^ of protein.

The activities of chitinase (EC 3.2.1.14) and β-1.3-glucanase (EC 3.2.1.6) were determined according to Côrtes et al. [15] using colloidal chitin and laminarin as the substrates. The reducing sugar released from the substrate was measured by according to the method described by Miller [16]. One unit of enzyme activity (IU) was defined as the amount of enzyme necessary to release 1 mmol of reducing sugar.h^-1^.mg^-1^ of protein. The activity of phenylalanine ammonia-lyase (EC 4.3.1.5) was determined by the method of Alunni et al. [17] using phenylalanine as the substrate. The absorbance of the trans-cinnamic acid derivatives was recorded at 290 nm (Spectrum SP– 2000, Spectrum Shanghai Ltd). One unit of enzyme activity was defined as the amount of enzyme necessary to increase absorbance by one unit at 290 nm h^-1^.mg^-1^ of protein.

### Anatomical analyses

For the observation of *Trichoderma* spp., the collected roots were placed in 10% KOH for 10 minutes, then in 2% HCl for 3 hours, and stained with Trypan blue. Subsequently, roots were sectioned into fragments of approximately one centimeter and mounted on histological slides with acidified glycerin. Samples of leaves from the third node below the shoot apex were used for all leaf analyses. The middle third of the central leaflet was used for standardization. For the detachment of epidermis, samples were exposed to a hydrogen peroxide and acetic acid solution (1:1, v:v), and incubated in an oven at 60°C for 48 hours [18]. Subsequently, detached epidermal fragments were stained with 1% safranin, mounted on histological slides with 50% glycerin, and observed under a light microscope at a magnification of 100x. Stomata on both sides of the leaf epidermis were counted in 10 fields (field area was calculated in μm^2^ and transformed to mm^2^) per repetition, taken randomly, with three replicates for each treatment.

For histological evaluation, leaf samples and roots (median region of the main root), were fixed in FAA 50 solution (formaldehyde 37–40%, acetic acid and 50% ethanol; 1:1:18, v:v:v) for 48 hours. Samples were transferred to 70% ethanol, dehydrated in an ethanol series, and embedded in Paraplast^®^ [19]. Cross sections (8–10 μm thick) were obtained on a Leica RM2245 rotary microtome and affixed to histological slides with Haupt’s adhesive [20]. After dewaxing, sections were hydrated using a descending ethanol series, stained with 0.3% Astra blue and 0.1% safranin (9:1, v:v), dehydrated using an ascending ethanol series, and mounted on histological slides with glass varnish [21]. All microscopic analyses were performed using a Leica DM 2000 microscope, coupled to a Leica EC3 digital camera. Quantitative analyses were performed with images taken from three replicates of each treatment using Leica LAS/EZ software version 3.3.0 image capture program (Leica Microsystems, Wetzlar, Germany). The results were statistically interpreted by analysis of variance using the software SISVAR 5.6, and by principal component analysis using the software PAST 3.

## Results

### Inoculation, plant growth and development

**Fig 1:**
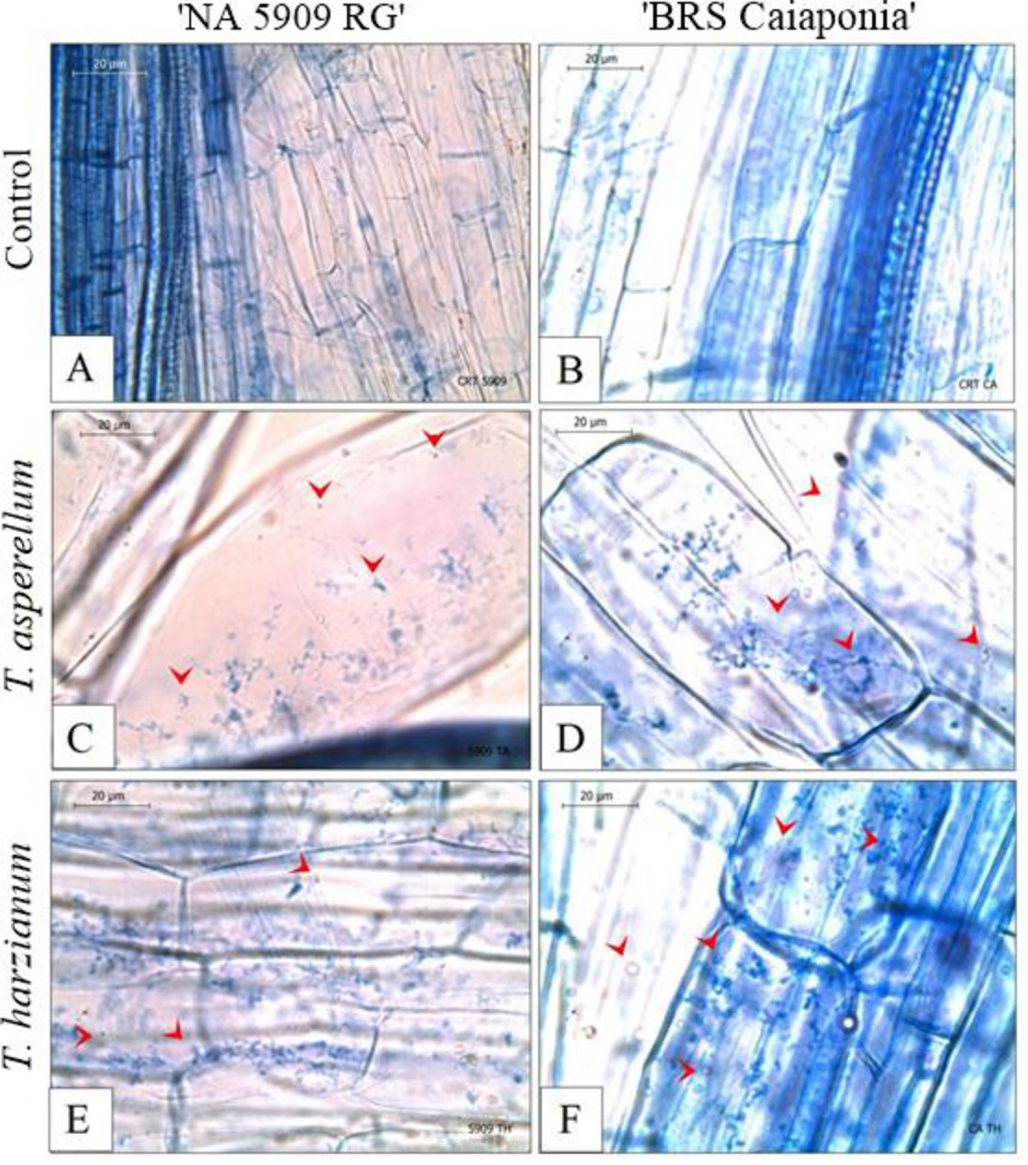
Parenchymatic cortical cells of the primary roots of soybean cultivars colonized by *Trichoderma* spp. Treatments: A) Uninoculated control of the cultivar NA 5909 RG; B) Uninoculated control of the cultivar BRSGO Caiaponia; C) *T*. *asperellum* on NA 5909 RG; D) *T*. *asperellum* on BRSGO Caiaponia; E) *T*. *harzianum* on NA 5909 RG; F) *T*. *harzianum* on BRSGO Caiaponia. Arrowheads: hyphae/spores.

**Fig 2:**
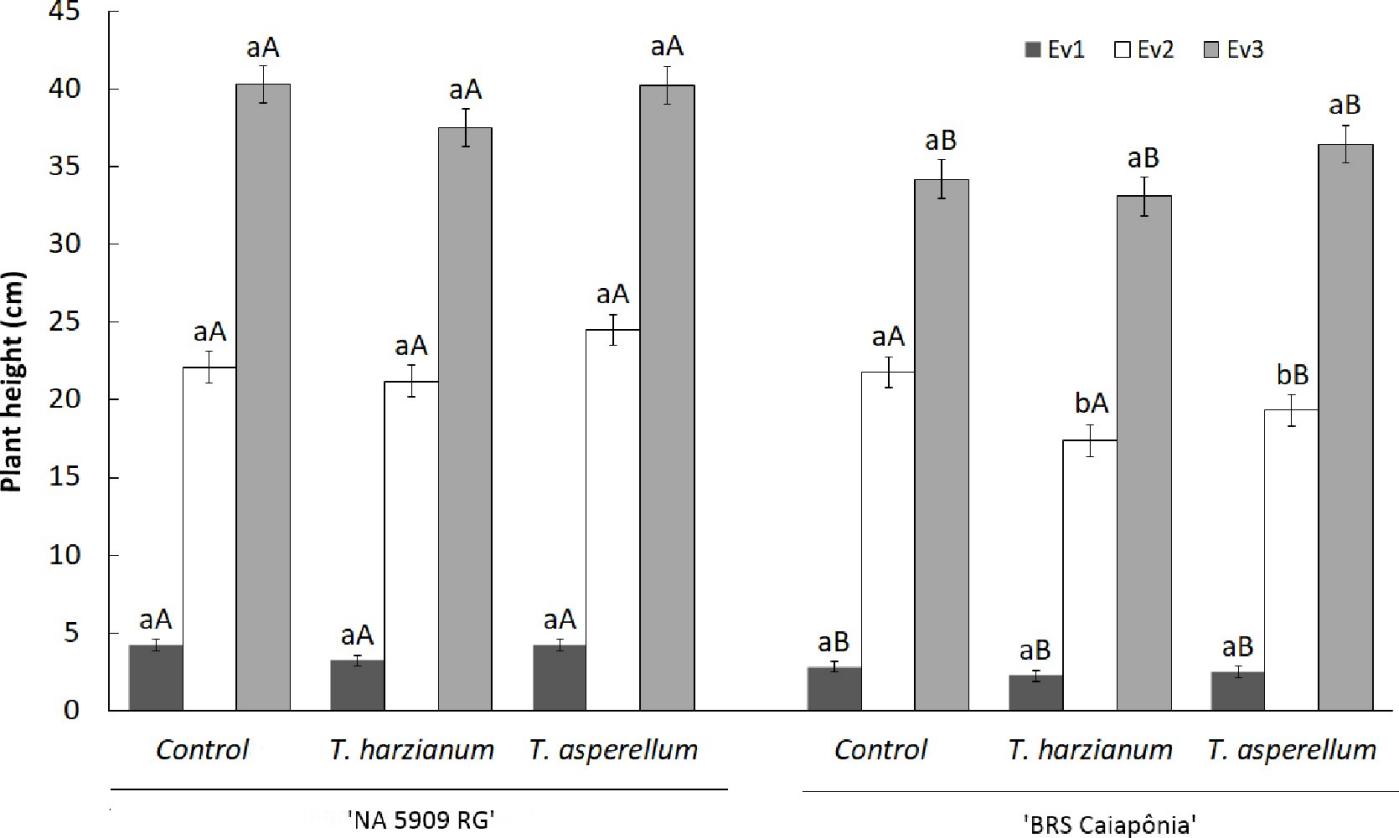
Evaluation of the height of soybean cultivars (NA 5909 RG and BRSGO Caiaponia) at 7 (Ev1 –dark gray), 14 (Ev2—white) and 21 (Ev3 –light gray) days after sowing, inoculated with two isolates of *Trichoderma* spp. (*T*. *asperellum*, *T*. *harzianum*). Bars signaled by the same lowercase letters do not differ among treatments, and uppercase letters do not differ between cultivars, by Tukey’s test at 5% probability.

*T*. *harzianum* and *T*. *asperellum* colonized the parenchyma cells of the cortical region in the roots of both soybean cultivars treated with the fungi (Fig 1C–1F), and colonization was not observed in the control treatment (Fig 1A and 1B). There was no visual difference in the observed amount of fungal colonization, but *T*. *harzianum* colonized more parenchyma cells in the roots of the analyzed plants than did *T*. *asperellum*. No differences in plant height were observed during plant development, except for BRSGO Caiaponia at 14 DAS treated with *T*. *asperellum* and *T*. *harzianum*, which were shorter than the other treatments at the same developmental stage (Fig 2). The fresh weight of analyzed plants also did not present differences among the treatments. In the comparative evaluation between cultivars, at 7 and 21 DAS the cultivar NA 5909 RG presented the largest plant sizes for all treatments. At 14 DAS only the *T*. *asperellum* treatment resulted in taller NA 5909 RG (Fig 2).

### Activity of defense-related enzymes

**Fig 3:**
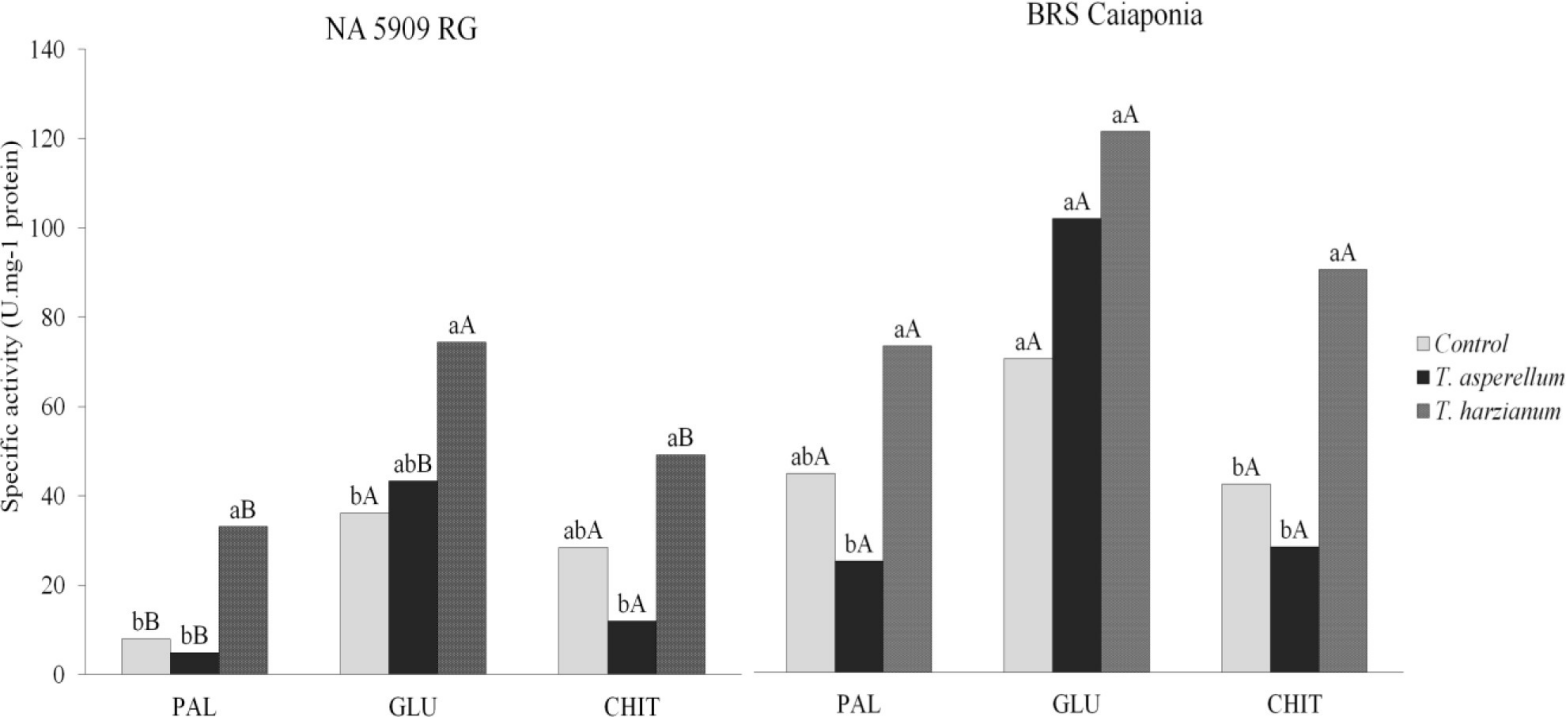
Activity of phenylalanine ammonia-lyases (PAL), β-1-3-glucanases (GLU) and chitinases (CHI) in soybean leaves from cultivars NA 5909 RG and BRSGO Caiaponia non treated or treated with *T*. *asperellum* or *T*. *harzianum*. Means followed by the same lowercase letters (effect of treatments), and uppercase (between cultivars) did not differ by Tukey’s test at 5% probability.

Overall, both cultivars showed increased activities of phenylalanine ammonia lyase (PAL), chitinase (CHI) and β-1,3-glucanase (GLU) after treatments with *Trichoderma* spp. when compared to untreated control (Fig 3). However, the average activities of all enzymes were higher in BRSGO Caiaponia than in NA5909RG. In relation to cultivar NA5909RG, the activities of PAL, GLU and CHI were significantly higher in the treatment with *T*. *harzianum* compared to untreated control; the highest activity was observed for GLU (Fig 3). In the cultivar BRSGO Caiaponia, PAL and CHIT activities after *T*. *harzianum* treatment were significantly higher than the untreated control and *T*. *asperellum* treatment (Fig 3). In this cultivar there were no significant differences in GLU activity between treatments and the untreated control.

### Anatomy of vegetative organs

**Fig 4:**
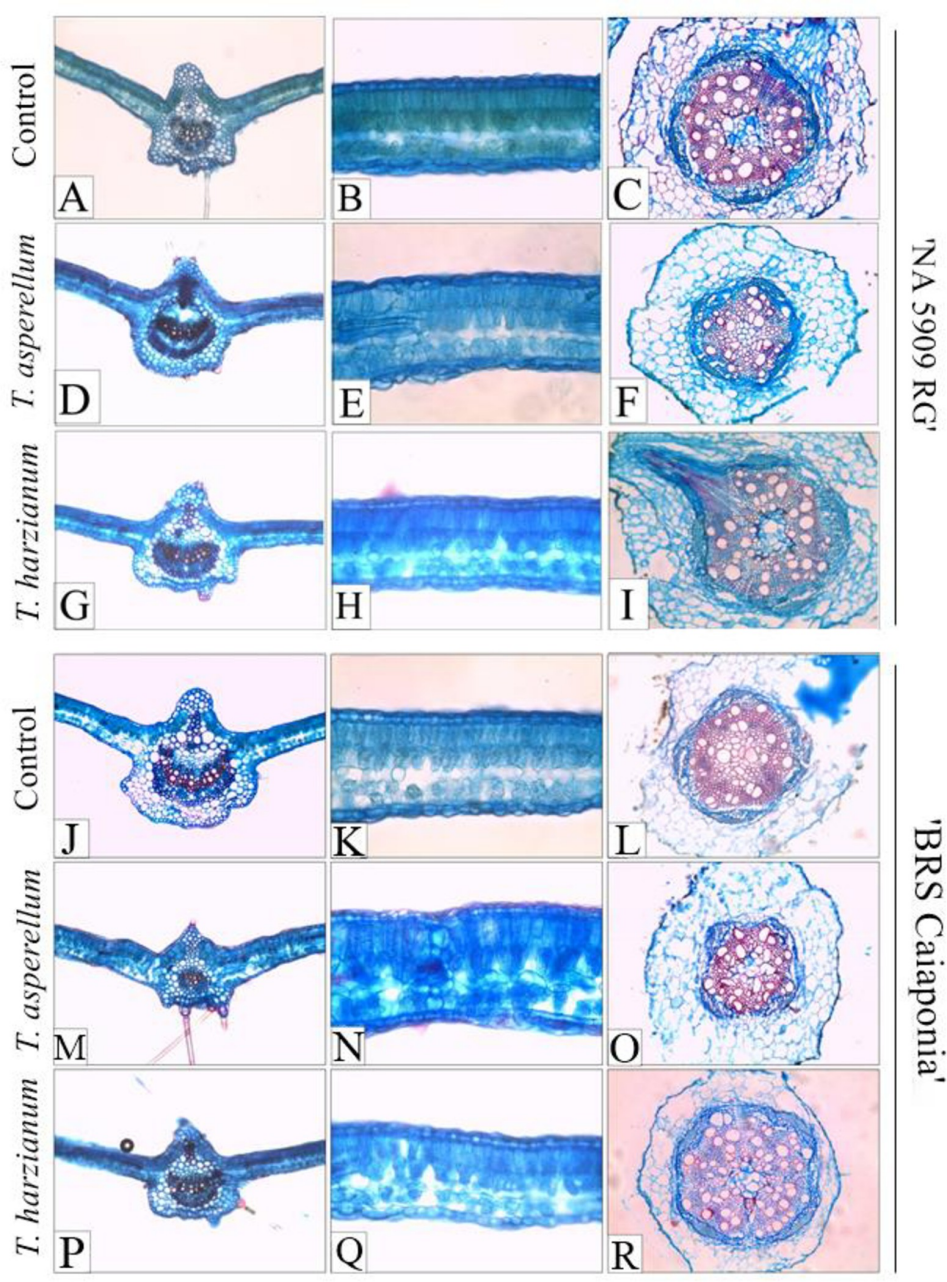
Cross sections of the leaf lamina of soybean cultivars NA 5909 RG (A-I) and BRSGO Caiaponia (J-R). Midrib—200 μm (A, D, G and J, M, P); Mesophyll, 100 μm (B, E, H and K, N, Q); Cross-sections of the root median region 200 μm (C, F, I and L, O, R). Treatments: Uninoculated control: A, B, C and J, K, L; *Trichoderma asperellum*: D, E, F and M, N, O; *Trichoderma harzianum*: G, H, I and P, Q, R.

The analyzed roots were in secondary growth. They presented epidermis, parenchymatic cortex, nonfunctional primary phloem, poorly developed secondary phloem, and prominent secondary xylem (Fig 4C, 4F, 4I, 4L, 4O and 4R). The vascular cambium between the secondary phloem and secondary xylem was not distinguishable. Within the structure, the primary xylem was observed, with four protoxylem poles and unignified metaxylem at the center, except for the BRSGO Caiaponia control (Fig 4C, 4F, 4I, 4L, 4O and 4R).

**Fig 5:**
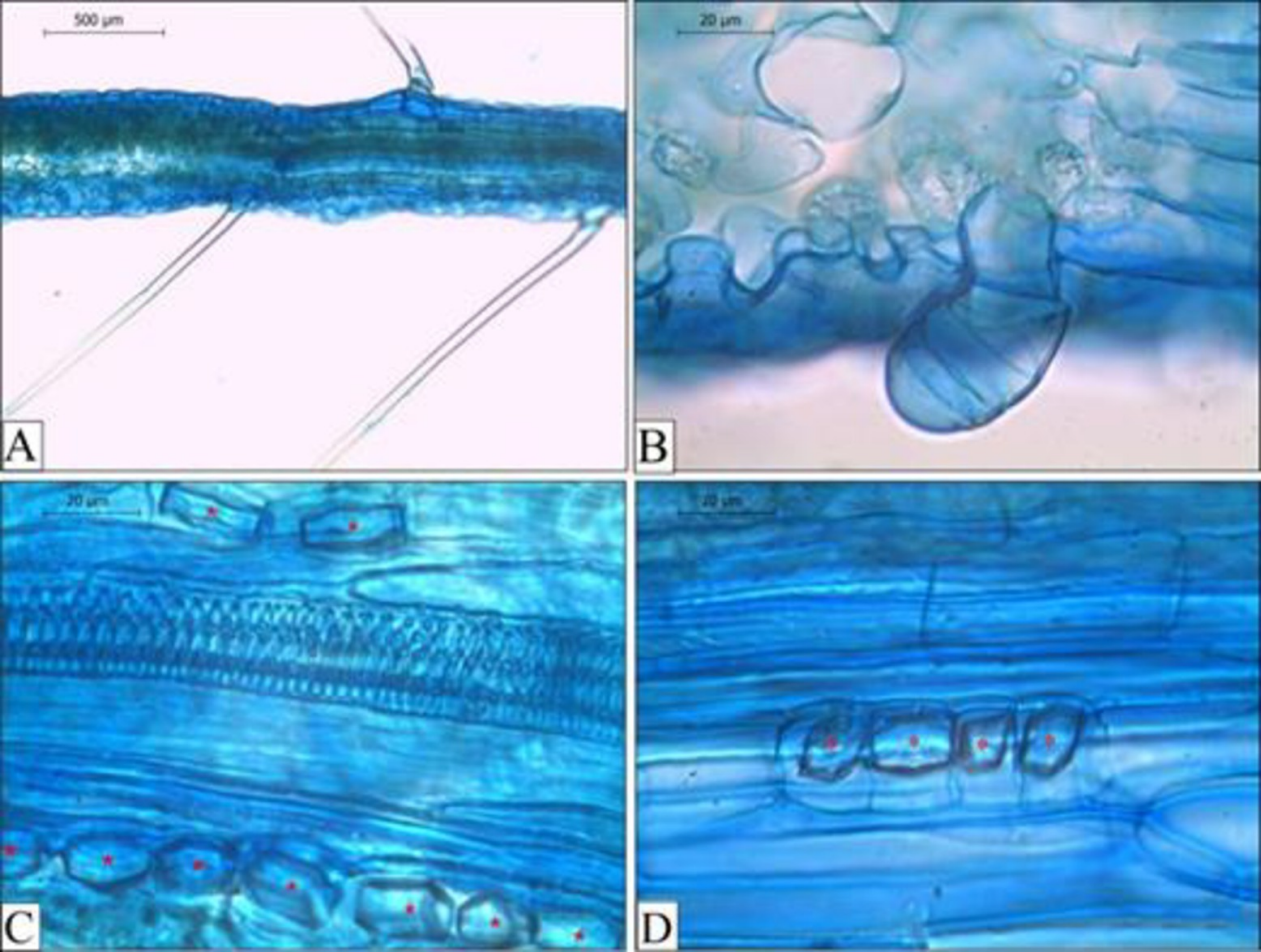
Leaf lamina of soybean cultivar NA 5909 RG, uninoculated (control). Cross sections: A) Tector trichome; B) Glandular trichome. Longitudinal sections: C) general view of minor veins with calcium oxalate crystals at the periphery; D) Detail of calcium oxalate crystals (*).

With regard to the anatomy of leaves, the mesophyll (ME) was dorsiventral, with two layered palisade parenchyma, and two to three layered spongy parenchyma, with cells of varying sizes and shapes, and wide intercellular spaces (Fig 4B, 4E, 4H, 4K, 4N and 4Q). Between the palisade and the spongy parenchyma, one layered paravenal parenchyma was observed in all treatments. The structural organization of the leaf mesophyll did not present alterations in relation to the control. In cross sections, the epidermis of the plants remained uniseriate on both surfaces, presenting cells of varying shapes and sizes (Fig 4A, 4B, 4D, 4E, 4G, 4H, 4J, 4K, 4M, 4N, 4P and 4Q). Tector trichomes were observed in all treatments, and consisted of a basal cell, an intermediate cell, and an apical elongated cell, of different dimensions (Fig 5A). Glandular trichomes were unisserated and claviform, composed of about five cells (Fig 5B).

**Fig 6:**
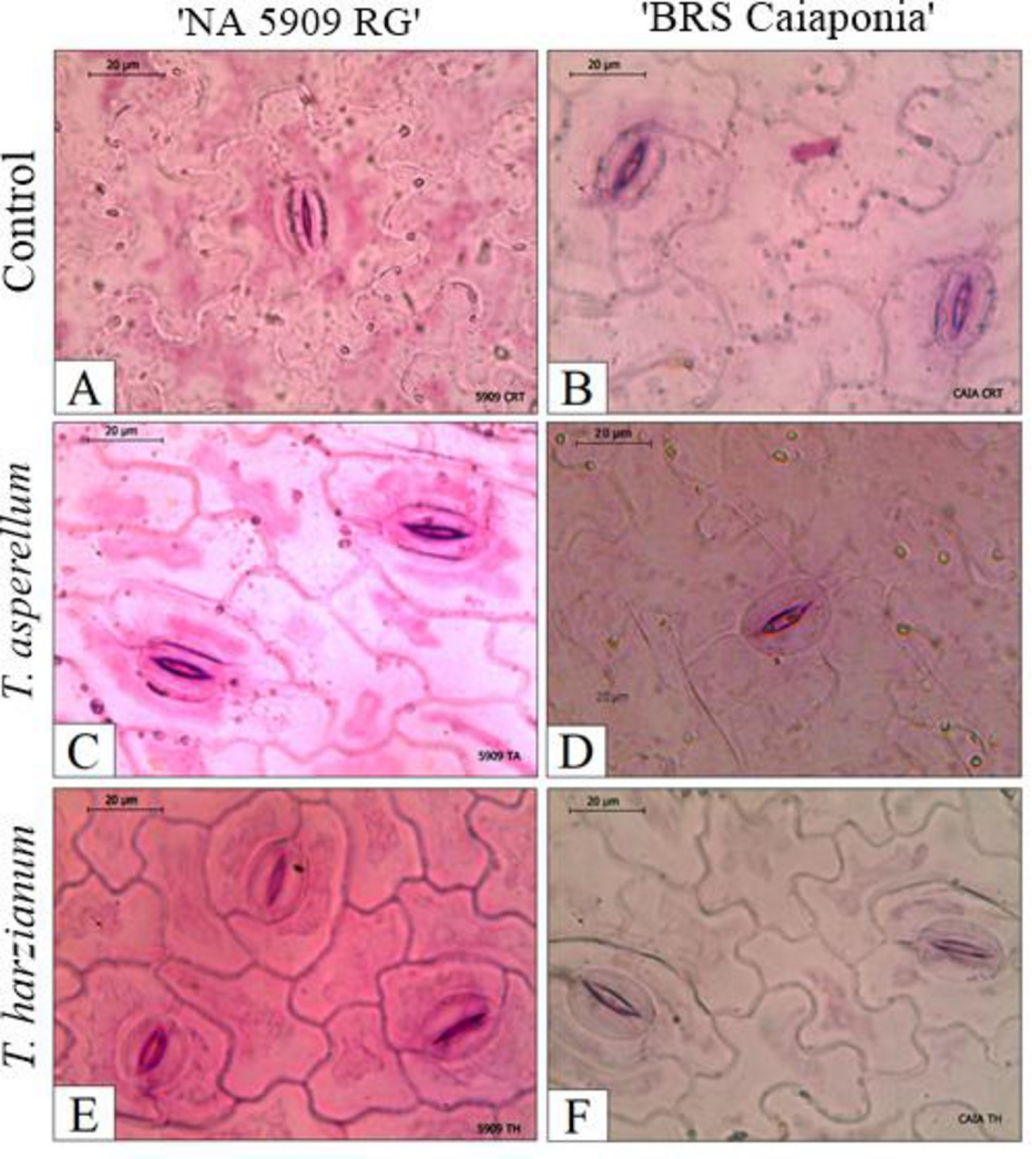
Detached epidermis from the abaxial surface of the leaves of soybean cultivars inoculated with *Trichoderma* spp. isolates, evidencing the paracytic stomata. Treatments: A) Uninoculated control on NA 5909 RG; B) Uninoculated control on BRSGO Caiaponia; C) *T*. *asperellum* on NA 5909 RG; D) *T*. *asperellum* on BRSGO Caiaponia; E) *T*. *harzianum* on NA 5909 RG; F) *T*. *harzianum* on BRSGO Caiaponia.

The median veins had a rounded contour on the abaxial surface and slightly sharp contour on the adaxial surface. A subepidermal collenchyma was observed on both surfaces, and a narrow layer of cortical parenchyma was located around the vascular tissues. Vascular tissues were constituted by a single beam with adaxial-facing xylem and abaxial-facing phloem arranged collaterally. Pericyclic fibers were observed surrounding the phloem and xylem (Fig 4A, 4D, 4G, 4J, 4M and 4P). Calcium oxalate crystals occurred in the cells of the parenchymatic bundle sheath (Fig 5C and 5D). In frontal view, the abaxial epidermal cells of the uninoculated control plants showed thin, sinuous anticlinal walls (Fig 5A and 5B). In frontal view, the epidermis showed different sinuosity of the anticlinal walls in comparison to the uninoculated control and the treatments with the fungi, especially in the cultivar NA 5909 RG (Fig 5C and 5D). The paracytic stomata (Fig 6) were present on both sides of the leaf lamina.

### Histometric analysis

Histometric and cytometric analyses of roots and leaves are described in Tables 1 and 2. In the roots, the treatments with *T*. *asperellum* did not affect any of the evaluated parameters in the cultivars NA 5909 GR and BRSGO Caiaponia when compared to the respective controls. Treatment with *T*. *harzianum* resulted in increased diameter of the xylem vessel elements in BRSGO Caiaponia but did not affect the cortex thickness or vascular cylinder diameter of the roots of either cultivar (Table 1).

**Table 1:** Histometric and cytometric analyses of roots of two soybean cultivars (NA 5909 RG and BRSGO Caiaponia) treated with *Trichoderma asperellum* and *Trichoderma harzianum*.

	Treatments		Roots		
			RC	VC	XyR
Cultivars	NA 5909 RG	**Control**	227.87abA	832.16aB	51.18aA
		***T*. *asperellum***	200.47bB	953.78aA	57.63aA
		***T*. *harzianum***	286.84aA	747.66aA	65.18aA
	BRSGO Caiaponia	**Control**	200.84abA	486.75aA	44.33bA
		***T*. *asperellum***	269.27aA	549.76aB	48.34abA
		***T*. *harzianum***	151.39bB	612.36aA	59.15aA
	CV (%)		21.83	19.18	15.27

In the columns, lowercase letters represent the comparisons between controls and treatments for each cultivar; upper letters case represent the paired comparisons between controls and treatments of both cultivars. Means followed by the same letters do not differ from each other by Tukey’s test at 5% probability. RC: thickness of the root cortex (μm); VC: mean diameter of the vascular cylinder (μm); XyR: mean diameter of the vessel elements in the xylem of roots (μm).

**Table 2:** Histometric and cytometric analyses of leaves of two soybean cultivars (NA 5909 RG and BRSGO Caiaponia) treated with *Trichoderma asperellum* and *Trichoderma harzianum*.

	Treatments		Leaves							
			XyL	ME	PP	SP	SDAdE	SDAbE	AdE	AbE
Cultivars	NA 5909 RG	**Control**	232.10aA	111.37bA	45.72aB	65.65bA	6.00	132.45bA	8.31bB	9.70
		***T*. *asperellum***	217.68aA	150.81aA	50.43aA	112.47aA	8.66	190.40abA	13.19aA	11.41
		***T*. *harzianum***	330.13aA	117.64bA	51.93aA	65.71bA	9.00	426.33aA	13.82aA	10.98
	BRSGO Caiaponia	**Control**	197.49aA	113.58aA	61.98aA	51.59aA	5.66	109.40aA	11.67aA	10.42
		***T*. *asperellum***	118.36aB	88.60aB	43.38bA	45.21aB	11.33	149.01aA	10.95aA	9.48
		***T*. *harzianum***	156.50aB	104.42aA	44.29bA	60.13aA	12.00	223.51aA	8.48aB	9.85
	CV (%)		24.38	13.18	15.22	22.17	7.59	41.73	17.62	14.27

In the columns, lowercase letters represent the comparisons between controls and treatments for each cultivar; upper letters case represent the paired comparisons between controls and treatments of both cultivars. Means followed by the same letters do not differ from each other by Tukey’s test at 5% probability. XyL: mean diameter of vessel elements in the xylem of leaves (μm); ME: mean thickness of the leaf mesophyll (μm); PP: mean thickness of the palisade parenchyma (μm); SP: mean height of the spongy parenchyma (μm); SDAdE: stomatal density of the adaxial epidermis (stomata per mm^2^); SDAbE: stomatal density of the abaxial epidermis (stomata per mm^2^); AdE: thickness of the adaxial epidermis (μm); AbE: thickness of the abaxial epidermis (μm).

When comparing the treatments with *T*. *asperellum* and *T*. *harzianum*, both for each cultivar and between cultivars, it was observed that cortex thickness in NA 5909 RG was smaller than that in BRSGO Caiaponia treated with *T*. *asperellum*, whereas for NA 5909 GR, it was larger than in BRSGO Caiaponia treated with *T*. *harzianum*, despite the similarity between the controls. In the comparison between controls of both cultivars, it was observed that the diameter of the vascular cylinder was naturally larger in NA 5909 GR than in BRSGO Caiaponia; this difference was maintained when comparing the cultivars treated with *T*. *asperellum*. The treatments with *T*. *harzianum* on NA 5909 GR and BRSGO Caiaponia resulted in statistically equal vascular cylinder diameters, despite the differences between the controls (Table 1).

In the leaves, the treatment with *T*. *asperellum* on NA 5909 RG resulted in increased thickness of the mesophyll and of the spongy parenchyma, both in relation to the control and the treatment with *T*. *harzianum*. *T*. *asperellum* also caused the adaxial epidermis of NA 5909 RG to grow thicker than that of the control, but did not differ statistically from the treatment with *T*. *harzianum* (Table 2). As an exclusive anatomical trait induced by *T*. *harzianum* on NA 5909 RG, increased stomatal density on the abaxial leaf surface was observed when compared to the control. In BRSGO Caiaponia, both treatments with *T*. *asperellum* and *T*. *harzianum* caused a decrease in thickness of the palisade parenchyma, and no other parameters were changed by such treatments when compared to the control (Table 2).

When comparing the controls of the cultivars, it was observed that the palisade parenchyma and the adaxial epidermis were naturally thicker in BRSGO Caiaponia than in NA 5909 RG (Table 2). However, treatments with *T*. *asperellum* and *T*. *harzianum* tended to decrease such parameters in BRSGO Caiaponia and to increase them in NA 5909 RG. Thus, the comparison between NA 5909 RG and BRSGO Caiaponia treated with *T*. *asperellum* and with *T*. *harzianum* revealed statistically equal values ​​for the thickness of the palisade parenchyma. As far as the thickness of the adaxial epidermis is concerned, both cultivars treated with *T*. *asperellum* had equally thick adaxial epidermises, while the treatment with *T*. *harzianum* caused BRSGO Caiaponia to have a thinner adaxial epidermis when compared to NA 5909 RG, contrary to what was observed for the controls (Table 2). Other parameters such as the diameter of the vessel elements in the xylem, the thickness of the mesophyll and that of the spongy parenchyma, were equal between controls, but the treatments with *T*. *asperellum* caused these parameters to decrease in BRSGO Caiaponia and to increase in NA 5909 RG (Table 2). This resulted in significantly different values under such conditions. Similar behavior was observed for the treatment with *T*. *harzianum* in which NA 5909 RG presented a larger diameter of the vessel elements in the xylem compared to BRSGO Caiaponia, despite the similarity between the controls (Table 2).

**Fig 7:**
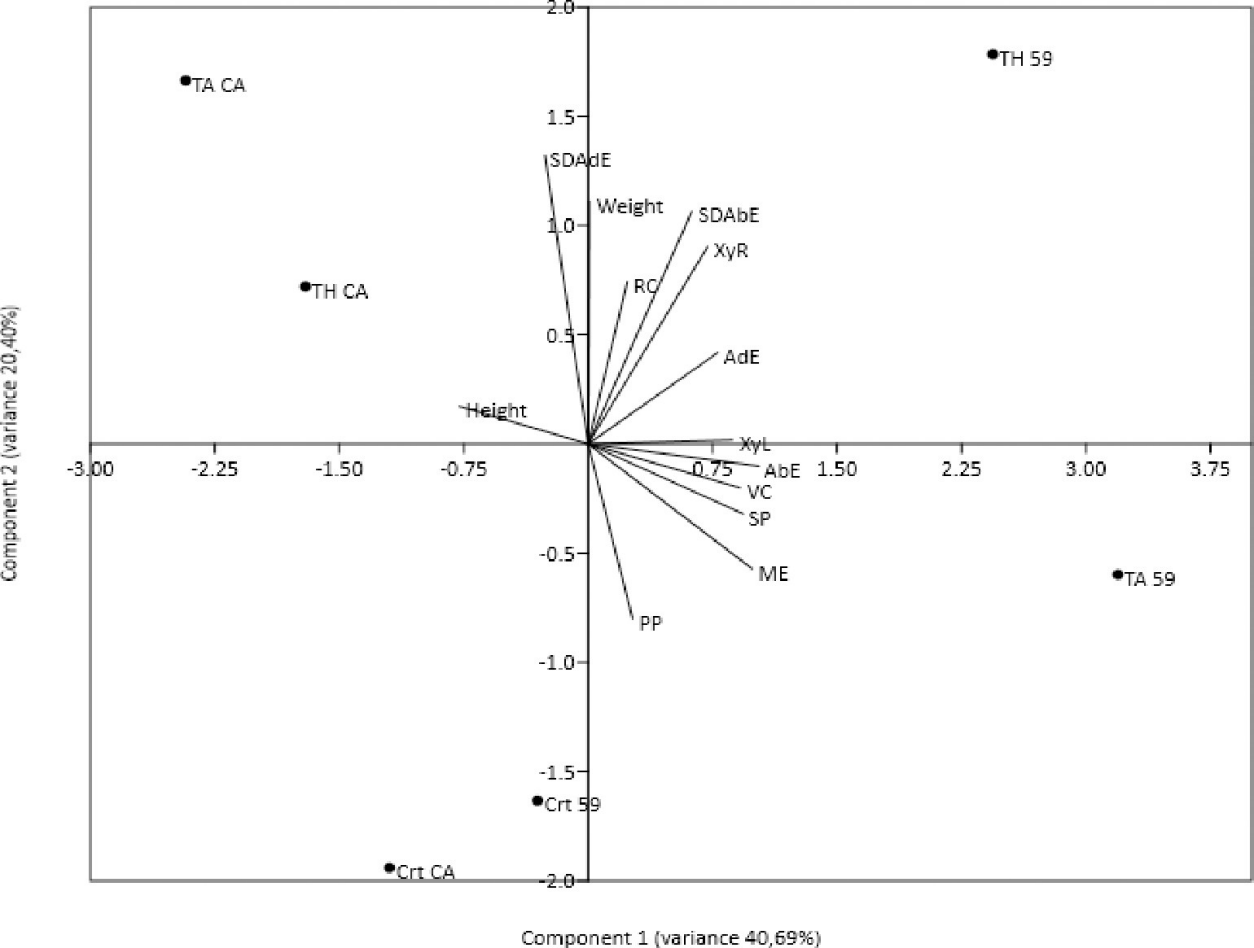
Ordering diagram by principal component analysis of soybean cultivars treated with *Trichoderma* spp. isolates. Variables of soybean growth: height 21 days after sowing and weight of the plant. Anatomical data: RC: thickness of the root cortex (μm); VC: mean diameter of the vascular cylinder (μm); XyR: mean diameter of the vessel elements in the xylem of roots (μm); XyL: mean diameter of vessel elements in the xylem of leaves (μm); ME: mean thickness of the leaf mesophyll (μm); PP: mean thickness of the palisade parenchyma (μm); SP: mean height of the spongy parenchyma (μm); SDAdE: stomatal density of the adaxial epidermis (stomata per mm^2^); SDAbE: stomatal density of the abaxial epidermis (stomata per mm^2^); AdE: thickness of the adaxial epidermis (μm); AbE: thickness of the abaxial epidermis (μm).

The principal component analysis performed with the anatomical data and soybean growth variables explained 61.09% of the total data variability, with 40.69% explained by the first component and 20.40% explained by the second (Fig 7). It was found that the plant height was inversely related to the diameter of the vascular cylinder (VC), the greater the height, the smaller the diameter of the vascular cylinder of the root. The highest values for the variables were: fresh weight of the plant, thickness of the adaxial epidermis (AdE) of leaves, stomatal density of the abaxial epidermis (SDAbE), thickness of the root cortex (RC), and mean width of xylem root cells (XyR), which were related to the cultivar NA 5909 RG treated with *T*. *harzianum*, having a positive correlation. On the other hand, the following variables: thickness of the abaxial epidermis (AbE), palisade parenchyma (PP) and spongy parenchyma (SP), mesophyll (ME) and root vascular cylinder (VC) were related to the cultivar NA 5909 RG treated with *T*. *asperellum*. The cultivar BRSGO Caiaponia was related to the height of the plants and the stomatal density of the adaxial epidermis (SDAdE). Controls were isolated in one quadrant, showing inverse correlation with the treatment with *T*. *harzianum* on cultivar NA 5909 RG (Fig 7).

## Discussion

Anatomical studies of plants are an important tool in the evaluation of the effects induced by plant growth promoting organisms (PGPMs) or biological control agents, such as bacteria and fungi. This work describes some relevant anatomical aspects during the interaction of *T*. *harzianum* and *T*. *asperellum* with two soybean cultivars (BRSGO Caiaponia and NA 5909 RG). Other papers using *Trichoderma* spp. in association with other PGPMs, such as bacteria and fungi, in the morphology and physiology of soybean plants have already been described [9, 10]. Yadav et al. [9] reported an improvement in morphological and physiological parameters in soybean plants after treatment of seeds with a combination of *Glomus mosseae*, *Acaulospora laevis*, *Trichoderma viride* and *Bradyrhizobium japonicum*. Paradiso et al. [10] also reported morphological changes in soybean plants after treatment with a mixture of 14 bacteria, yeasts and 12 beneficial fungal species (*Mycorrhizae* and *Trichoderma* spp). However, in these works the authors used a mix of PGPMs associated with isolates of *Trichoderma*.

In general, the treatments of both cultivars with *T*. *harzianum* and *T*. *asperellum* did not affect plant height, except for BRSGO Caiaponia, which were smaller than the control after 14 days of sowing (Ev. 2). These data indicated that the fungi may take a few days to begin their interaction with the plants and that BRSGO Caiaponia is more sensitive to the presence of the fungi when compared to NA 5909 RG. Biological products are made up of living organisms that must survive, colonize and multiply in the plant or in the environment in which they are applied. Thus, the efficiency of bioproducts can be directly affected by local biotic factors and require some time to establish interactions with plants to become effective against phytopathogens [22]. In fact, the increased activity of defense-related enzymes observed in both soybean cultivars illustrate that the metabolic apparatus of the plants is energetically redirected toward the mediation of plant-fungus interactions, as is true for most plants [23]. Such interactions demand energy from the plant, which must adapt both anatomically and physiologically for the successful establishment of the fungi [23], which could explain why BRSGO Caiaponia plants presented the highest degrees of enzymatic activity alterations and were smaller after 14 days of sowing.

In order to better evaluate the reprogramming of plant metabolism and structure due to the establishment of plant-fungus interactions, anatomical traits are reliable sources of information, and this is also true for other plant-organism interactions such as galls, environmental changes and interaction with pathogens [24–28]. In this study, both tector and glandular claviform trichomes were recorded in *G*. *max*, corroborating previous reports for such varieties of trichomes in the Fabaceae [25–28]. Similar to what has been reported in previous studies, trichomes were generally observed near the regions of the veins on both sides of the foliolar lamina, especially at the abaxial face of both cultivars. Trichomes play important roles in the context of plant ecology, and aspects related to their distribution and content may influence plant adaptability to a great extent. There are several functions of trichomes in plants, and tector trichomes can prevent undue loss of water [29, 30]. Another possible function, that of the cover trichomes, is in defense against pathogens [31, 32].

Anatomical traits other than trichomes may also indicate plant adaptability to a variety of stimuli. For example, studies using living organisms and/or plant extracts as plant treatments may potentialize plant growth, as well as induce activation of the plant's defense mechanisms against pathogens [28, 31]. Either way, several studies have reported anatomical changes in plant organs that are accompanied by adaptive responses. In this work, changes in some anatomical variables of soybean plants treated with *Trichoderma* spp. indicated that variables relate to the activation of plant defense mechanisms, as clearly demonstrated by increased PAL and GLU activities in NA 5909 RG and CHIT in BRS Caiaponia, and that the cultivars adapt differently to the fungi isolates, as *T*. *harzianum* induces the highest activities when compared to *T*. *asperellum*.

Stomata were found on both sides of the foliolar lamina. Amphistomatic leaf is a common characteristic in several genera of plants, mainly in cultivated plants, due to the exposure to high levels of light. As such, this characteristic allows greater conductance of CO_2_ and consequently, a higher photosynthetic rate [33–35]. Increased stomatal density on leaves as a response to treatments with *Trichoderma* spp. has been previously reported for rice cultivars [33], where the inoculation of *T*. *asperellum* increased the stomatal density by 23% and 69% in relation to the control plants. Such an increase was reflected in the growth of the plants, which showed improved physiological performance when compared to untreated plants [33]. Soybean cultivars grown under the same environmental conditions reacted differently to the treatments with the fungi, with the NA 5909 RG cultivar most responsive to the presence of *T*. *harzianum*, in terms of stomatal density. As is true for rice plants [33], increased stomatal density is a desirable anatomical feature in plants that may perform better under stressful conditions such as increased light irradiance and lack of water; this characteristic is observed for other drought-resistant plants [36–40]. Additionally, *Trichoderma* spp. induced the differentiation of a thicker adaxial epidermis in the NA 5909 RG cultivar, and this is also a common feature of highly light-adapted plants [8, 33], thus corroborating the hypothesis that *Trichoderma* spp. help plants to perform better under harsh conditions by ensuring increased photosynthetic rates and/or by inducing drought resistance.

Further evidence to support this hypothesis is observed in NA 5909 RG treated with *T*. *asperellum*, which showed increased thickness of the mesophyll and of the spongy parenchyma, a factor that directly relates to the photosynthetic apparatus of the plants, and has been shown to respond to different environmental conditions [8, 33]. On the other hand, the BRSGO Caiaponia cultivar treated with *T*. *harzianum* showed an increased diameter in vessel elements in the xylem of roots, which increases their capacity for water uptake and transportation [39, 40], and is thus a desirable anatomical feature that is likely of adaptive importance to the plants. BRSGO Caiaponia reacted negatively to the presence of *Trichoderma* spp. by decreasing the thickness of the palisade parenchyma, which may result in lower photosynthetic capacity. Nevertheless, further studies should address this response to better understand the significance of such a change from the perspective of plant performance. In general, the anatomical characteristics of plants described herein match those of plants adapted to greater light intensity and greater efficiency in water use [40, 41]. It should be noted that the cultivars showed similar growth performance despite the different anatomical arrangements influenced by *Trichoderma* species. As the changes are observed principally in the leaves, while fungal colonization occurs in the roots, it is plausible to affirm that the treatments with *T*. *asperellum* and *T*. *harzianum* have a systemic effect on the plants.

The interaction between *Trichoderma* spp. and the cultivar NA 5909 RG resulted in an increase in the following variables: stomatal index at the abaxial leaf surface (SDAbE), thickness of the root cortex (RC), thickness of adaxial epidermis (AdE), mean diameter of the vascular cylinder (VC), thickness of the mesophyll (ME) and thickness of the spongy parenchyma (SP). These factors may result in increased photosynthetic efficiency through different pathways, which would result in a positive carbon shed, especially when the plant is exposed to certain types of stress, which provides evidence that plants have different metabolic response options available to them. In addition, cultivar NA 5909 RG was generally more responsive to the treatments used in this study, especially regarding leaf parameters, than was cultivar BRSGO Caiaponia. Finally, when comparing the two cultivars in general, leaf parameters tended to increase in NA 5909 RG while they decreased in BRSGO Caiaponia.

*Trichoderma* species affected soybean plants in different ways, resulting in different anatomical changes, but with similar overall responses. *T*. *asperellum* promoted anatomical changes in the diameter of the vascular cylinder, and diameter of vessel elements in the xylem of leaves, mesophyll, and spongy parenchyma. Furthermore *T*. *harzianum* was able to promote anatomical changes in adaxial epidermis thickness and stomatal density of the abaxial epidermis. The cultivar NA 5909 RG responded with more desirable anatomical changes in plants, for better performance against adverse conditions and pathogens.

## Conclusion

Anatomical studies are suggested as an important tool in assessing the effects induced by biological control agents, the market for which is gaining prominence, with concomitant necessity to increase the understanding of interaction mechanisms. This work describes relevant anatomical aspects for the characterization of soybean plants and their interaction with beneficial microorganisms. Thus, the variables analyzed herein can be taken as reliable anatomical markers that evidence the beneficial effects of interactions between fungi and plants. This will facilitate understanding the pathways that control biochemical and molecular changes, as well as possible strategies to maximize the positive effects of biostimulators in the field.
